# Explicit and Implicit Emotion Processing: The Role of Spatial Frequencies in a Case Study of Right Capsulo–Thalamic Damage

**DOI:** 10.3390/jintelligence14040060

**Published:** 2026-04-03

**Authors:** Vincenza Tommasi, Caterina Padulo, Giulia Prete, Antonio Leo, Alessandra Franco, Tatiana De Francesco, Maria Rosaria Viva, Luca Tommasi, Giuliana Lucci, Chiara Valeria Marinelli

**Affiliations:** 1 Cognitive and Affective Neuroscience Lab, Department of Humanities, Letters, Cultural Heritage, Education Sciences University of Foggia, 71121 Foggia, Italy; vincenza.tommasi@unifg.it; 2 Department of Humanities, University of Naples “Federico II”, 80133 Nales, Italy; caterina.padulo@unina.it; 3 Department of Psychology, “G. d’Annunzio” University of Chieti-Pescara, 66100 Chieti, Italy; giulia.prete@unich.it (G.P.);; 4 Santa Chiara Rehabilitation Institute, 73100 Lecce, Italy; 5 Independent Researcher, 00161 Rome, Italy; giiuliana.lucci@gmail.com

**Keywords:** hybrid faces, implicit emotion processing, capsulo–thalamic pathway, friendliness judgements, dual-route of emotion processing

## Abstract

This study examined the interaction between spatial frequencies and emotion processing using tachistoscopic presentations of emotional faces, in a patient with right capsulo–thalamic damage and a matched control group (N = 3). Emotional (happy, angry and sad) and neutral faces were presented in one of two ways: broadband emotional images and hybrid faces, which were created by superimposing emotional Low Spatial Frequencies (LSFs) to the High Spatial Frequencies (HSFs) of the same identity with a neutral expression, resulting in a subliminal presentation of the emotional content. According to LeDoux’s dual-route model, which suggests a cortical–conscious emotional analysis and subcortical–unconscious emotional processing, we expected healthy participants to show different variations in friendliness ratings compared with the case study patient. In particular, we hypothesized that while healthy participants should show friendliness ratings varying consistently with the facial expressions for both unfiltered (conscious) and filtered (unconscious) stimuli, reflecting the efficiency of both routes, the patient should show a selective deficit in the unfiltered condition due to the disruption of the thalamo–cortical connections. The results showed that healthy controls evaluated emotions consistently across both conditions. Notably, there were no significant differences between the case study patient and the control group for hybrid faces, suggesting that the “hidden” LSF successfully activated the intact subcortical route. However, significant differences emerged for unfiltered stimuli: the case study patient was able to distinguish between positive and negative valence, but she failed to discriminate between negative emotions. This finding suggests that the fine-grained differentiation of negative emotions requires an intact cortical analysis, mediated by the internal capsule.

## 1. Introduction

Emotions represent psychobiological changes in both external and internal states that can be referred to external or internal stimuli. They are considered adaptive manifestations of human behavior (Darwin, 1872) that should facilitate survival and affect decisions. Emotional processing can be categorized as either explicit or implicit, depending on the cognitive processes required (Celeghin et al., 2020).

### 1.1. Spatial Frequencies and Emotion Processing

The distinction between explicit and implicit processing of stimuli, including emotional stimuli, is particularly relevant when considering the role of spatial filtering in visual perception. The cognitive system analyzes a visual stimulus by breaking it down into spatial frequencies, which are bands of light and dark that differ in luminance and contrast (Campbell & Robson, 1968). This process allows for the detection of both threats and opportunities, even when the visual conditions are perceptually impoverished (Seligman, 1971; Öhman & Mineka, 2001). High spatial frequencies (HSFs) correspond to grids with narrow, tightly packed light and dark bands. They convey detailed information about an image. In contrast, Low Spatial Frequencies (LSFs) correspond to wide and loosely packed bands and carry coarse, general information about an image (Schyns & Oliva, 1999; Vuilleumier et al., 2003). It is accepted that in the visual system, global analysis based on LSFs occurs earlier than local analysis based on HSFs (Le Grand et al., 2004), and a number of studies support the crucial role of LSFs in emotion processing (Vuilleumier et al., 2003; Prete et al., 2015a, 2015b, 2018; Prete & Tommasi, 2018; Tommasi et al., 2021; Schyns & Oliva, 1999; Sergent, 1985).

In this view, explicit emotions, consciously perceived and processed, are conveyed by unfiltered or unmanipulated images, meaning they involve deliberate cognitive appraisal and awareness of the emotional state. Conversely, implicit emotions can be conveyed by using a specific spatial filtering, with LSFs potentially being able to elicit an emotional processing that occurs without conscious awareness or direct cognitive control (Laeng et al., 2010). They are frequently triggered by subliminal or unacknowledged stimuli and processed through more automatic neural pathways.

### 1.2. The Dual-Route Model and Modern Perspectives

This distinction between explicit and implicit processing of visual stimuli is aligned with LeDoux’s dual-route model of the “high road” and “low road” (LeDoux, 2003).

The “high road” is a cortical, slow pathway providing experiential analysis and conscious response. The “low road” is a subcortical pathway involving the colliculo–pulvino–amygdalar circuit, which produces rapid amygdala activation and sensorimotor responses that are not necessarily consistent with the specific situation (Cunningham et al., 2004). While this formulation was instrumental in identifying the rapid subcortical pathway, modern neuroscience has moved toward a more nuanced view of distributed networks. The “Multiroad Model” (Pessoa & Adolphs, 2010) suggests that rapid fear detection might be mediated by multiple shortcuts within the visual cortex that link directly to the amygdala, proposing that cortical involvement is necessary even for fast responses. Despite these advancements in distributed network theories, the existence of a dedicated subcortical route for pre-attentive threat processing remains a subject of intense empirical support (Gainotti, 2012).

Architectural evidence provided by Dynamic Causal Modeling (DCM) demonstrates that a neuronal architecture incorporating this subcortical connection (Pulvinar to Amygdala) best explains brain activity during the earliest time periods of face processing (Garvert et al., 2014). Recent iEEG studies (Wang et al., 2023; Méndez-Bértolo et al., 2016) have shown that the human amygdala responds to invisible fearful faces at ultra-fast speeds (45–88 ms) independently of the visual cortex. This finding reinforces the existence of the evolutionarily ancient subcortical circuit for unconscious threat detection, even when compared with multiroad models. Consequently, while we acknowledge the involvement of broader distributed networks (LeDoux, 2012), the “low road” remains a biologically validated mechanism for the rapid detection of environmental danger, operating independently from slower cortical analysis. Furthermore, recent large-scale neuroimaging has validated the structural foundation of this system, demonstrating that the white matter fiber density of the pulvinar–amygdala pathway directly predicts an individual’s efficiency in recognizing fearful faces (McFadyen et al., 2019).

### 1.3. A Neuroanatomical Focus: The Internal Capsule of the Thalamus

Both pathways include the thalamus as the central station relay. The high road is a cortical pathway linking the thalamus to the cortex and subsequently relaying information to the amygdala. The low road is a subcortical pathway that directly conveys information from the thalamus to the amygdala with minimal cortical input. It produces a rapid and automatic sensorimotor response without awareness (Cunningham et al., 2004). Johnson (2005) suggested that the subcortical route operates most efficiently when potentially dangerous stimuli occur (see also LeDoux, 1996). However, different authors have also highlighted its involvement in smiling and positive-affect processing (Zald, 2003), suggesting that all emotional stimuli are processed by the subcortical route (Vuilleumier et al., 2003). Communication between the thalamus and cerebral cortex relies heavily on the internal capsule, a “V” or “boomerang” shaped band of white matter that carries information to and from the cerebral cortex (Emos & Agarwal, 2019).

The internal capsule is a complex structure where the posterior limb (PLIC) is associated with the alerting component of attention (Niogi et al., 2010; Fimm et al., 2001), while the anterior limb (ALIC) is associated with emotion, motivation, and cognition processing (Safadi et al., 2018). The ALIC is a site of convergence between the anterior thalamic radiations, implicated in emotional regulation, and the medial forebrain bundle (Coenen et al., 2012). Stimulation of the ALIC is associated with positive mood alterations (Okun et al., 2007; Machado et al., 2009), whereas its surgical ablation is thought to alleviate limbic dysfunction by interrupting the Papez circuit (Papez, 1937; Christmas et al., 2011; Mithani et al., 2020).

The role of the internal capsule and its involvement in both explicit and implicit emotion processing, particularly within the framework of LeDoux’s dual-route model, remains an area requiring further investigation. To address this, we developed an emotional recognition task utilizing a tachistoscopic divided visual field paradigm to manipulate types of processing (explicit–implicit) via spatial frequencies (hybrid stimuli).

The cortical pathway is sensitive to HSFs, while the subcortical pathway responds to LSFs via magnocellular neurons (Vuilleumier et al., 2003). The subcortical pathway transmits coarse information through the superior colliculus and pulvinar, bypassing time-consuming cortical routes (Tamietto & de Gelder, 2010), and shows higher sensitivity to invisible stimuli (de Gelder et al., 2011; Diano et al., 2017; Brooks et al., 2012; Axelrod et al., 2015; Juruena et al., 2010). Several studies have suggested that subcortical structures are more important for processing of emotional stimuli (e.g., Adolphs et al., 1994), and neuroimaging studies have shown cortical and subcortical activation during emotion recognition (Narumoto et al., 2000; Yip et al., 2004). Indeed, this road seems to be crucial for the early detection of threats or salient stimuli that demand immediate reactions before the brain’s cortical regions have fully analyzed the stimulus (Bar & Neta, 2006; Tippett et al., 2018).

Recent functional imaging, neuropsychological, and electrophysiological studies suggest that this system for face detection involves the superior colliculus, pulvinar, and amygdala as an integrated network (Johnson, 2005). Although early studies suggested that the subcortical route was specialized for threat (LeDoux, 1996), it was later shown to be involved in smiling and positive-affect processing (Zald, 2003), suggesting a subcortical involvement for all emotional stimuli (Vuilleumier et al., 2003). Direct evidence for this is provided by iEEG studies showing rapid amygdala activation for LSF fearful faces as early as 45 ms post-stimulus, functioning independently of conscious awareness (Wang et al., 2023).

### 1.4. Hemispheric Specialization of Emotion Processing

Differential spatial processing is supported by the brain’s hemispheric specialization. While both the left and the right hemisphere can process visuospatial information, the right hemisphere appears to be more efficient at global processing (LSFs), while the left hemisphere excels at local processing (HSFs; Sergent, 1982; Proverbio et al., 1997; Peyrin et al., 2003).

Debate continues regarding hemispheric specialization for emotional valence. The “right hemisphere hypothesis” argues that the right hemisphere is specialized in emotion processing, regardless of its valence (Gainotti, 1972; Levy et al., 1983). Conversely, the valence hypothesis proposes a differential specialization based on emotional valence: the right hemisphere is predominantly specialized for negative emotions processing, while the left hemisphere is more specialized in positive emotions processing (Davidson et al., 1987; Ehrlichman, 1987).

Taken together, these findings seem to suggest that the right hemisphere is better than the left hemisphere in emotional analysis (Gainotti, 2005) and that different spatial frequency bandwidths allow for different types of information processing, favoring one specific categorization task over the other (e.g., emotion vs identity recognition).

### 1.5. The Present Study

Our study involved a patient with internal capsular damage. Given that the internal capsule serves as the primary route connecting the thalamus to the cerebral cortex, its damage would specifically impair the proper functioning of the cortical “high road” processing. This unique case allows us to investigate the necessity of intact thalamo–cortical connections for both explicit and implicit emotion processing. The role of the internal capsule and its role in both explicit and implicit emotion processing has not been investigated yet.

While the unfiltered facial expressions served as explicit stimuli, hybrid stimuli were employed as implicit stimuli. Hybrid stimuli are created by the superimposition of two images that are filtered at different spatial frequencies. We used stimuli from Laeng et al. (2010), which are emotional faces filtered at LSFs and superimposed to the neutral expression of the same actor filtered at HSFs (Laeng et al., 2010). When an explicit evaluation was required, such as emotion identification, the authors found that participants categorized all stimuli as “neutral”, suggesting that they based their judgements on HSF-related information. When an implicit evaluation was required, that is, facial friendliness judgements, authors found that participants judged hybrid faces according to the LSF information. Thus, happy hybrid faces were judged as more friendly than neutral hybrid faces, which in turn were judged as more friendly than hybrid angry faces (Laeng et al., 2010). This suggests that the hidden emotional content was subliminally processed.

Explicit evaluation might involve conscious identification of an emotion (e.g., “What emotion is this face showing?”), while implicit evaluation could be assessed by an indirect measure, such as rating the “friendliness” of a face, even if the underlying emotion is not consciously recognized (Laeng et al., 2010, Prete et al., 2015a, 2015b, 2014; Tommasi et al., 2021). For the purpose of our study, “explicit” processing specifically refers to the “high road” activation necessary to the processing of HSFs. On the other hand, “implicit” processing, “low road” activation, is necessary to the processing of LSFs in which emotions are hidden below the threshold of awareness.

In our study, we also used facial friendliness judgments to obtain an indirect index of emotion processing to investigate the role of spatial filtering in conscious and nonconscious emotional perception (Laeng et al., 2010).

The main aim of this study was to evaluate, through a single-case design, the differential impact of subcortical and cortical neural pathways on the processing of facial stimuli, specifically concerning spatial frequency and affective content. By investigating the performance of patient R., who presents a focal lesion in the right internal capsule, compared with a matched control group, we had three clear objectives:

1. To assess the involvement of the thalamic internal capsule in emotion processing at different levels of awareness. We utilized the patient’s lesion as a natural model for the disruption of thalamo–cortical connectivity to complete this objective.
2. To verify hemispheric specialization for processing spatial frequencies, with a focus on local (HSFs) versus global (LSFs) information. Specifically, to verify how a right-sided subcortical disconnection affects hemispheric specialization for spatial frequencies.
3. To investigate hemispheric specialization for emotion processing. This was accomplished by comparing the patient’s performance across visual fields against the baseline of the control group.

Following LeDoux’s dual-route model, we hypothesized the following: (1) Healthy participants would show friendliness ratings (as indirect measures of emotion processing) varying consistently with the emotional facial expressions. Specifically, we expected happy faces to be judged as more friendly than neutral ones, and neutral faces as more friendly than angry ones. This pattern was predicted for both unfiltered and hybrid stimuli, reflecting the efficiency of both cortical and subcortical routes. (2) The patient R., due to her specific lesion in the thalamic internal capsule disrupting thalamo–cortical connections, would exhibit a selective impairment in processing explicit (unfiltered) emotional stimuli (cortical route). Specifically, we expected R. to show a deficit in explicit emotional evaluation for unfiltered faces, while potentially showing preserved performance for hybrid stimuli, where the subcortical LSF pathway remains intact.

## 2. Materials and Methods

### 2.1. Participants

R. is a 59-year-old right-handed woman who had a vascular stroke in the territory of the internal capsule in the right hemisphere when she was 52 years old. Written and informed consent to participate in this research was obtained, and the ethics committee of the Santa Chiara Rehabilitation Institute (Lecce), Italy, approved the study. R.’s postoperative MMSE (Mini Mental State Examination) corrected score was 26.74.

Depressive symptomatology was assessed using the Beck Depression Inventory (BDI; Beck et al., 1996), demonstrating no symptoms and a score of 12. Her laterality quotient is 66.6, according to the Edinburgh Handedness Inventory (Oldfield, 1971). One year after the stroke, the patient presented hypodensity regions in the right thalamic area and at the level of the caudate nucleus head and body; moreover, hyperdensity of the basal nucleus was present, particularly in the left hemisphere. R. had normal or corrected-to-normal vision, she had no psychiatric symptoms and she was tested at the Clinic (Italy; Santa Chiara Rehabilitation Institute, Lecce) during a session of physical and neuropsychological rehabilitation. She was unaware of the purpose of the study. The procedure was implemented in accordance with the code of ethics of the World Health Organization and with the Helsinki Declaration and approved by the Ethics Committee of Psychological Research of the Humanities Department of the University of Foggia (Prot. 3992/CEpsi n.143/25).

As a control sample, three healthy female participants were also recruited. Their mean age was 60.33 (±2.08), and their mean handedness score was 47.86 (±25.02) according to the Edinburgh Handedness Inventory (Oldfield, 1971). All participants had 13 years of education (high school diploma), matching the educational background of the patient, had normal or corrected-to-normal vision and no history of significant visual impairments that could affect emotion perception. Their MMSE mean corrected score was 28.66 (±0.06). A MMSE cut-off score of 26 is used to suggest possible global cognitive impairment and dementia according to indications provided by the AIFA (Italian Medicines Agency; https://www.aifa.gov.it/nota-85 (accessed on 15 December 2025); https://www.aifa.gov.it/documents/20142/1728086/nota-85.pdf (accessed on 15 December 2025)). Depressive symptomatology was measured using the Beck Depression Inventory (BDI; Beck et al., 1996), demonstrating no symptoms (score = 9.33 ± 4.16). Before the task, participants gave informed consent to take part in the study, they signed a consent to data processing, and after the end of the task, they were debriefed. The healthy participants were unaware of the purpose of the study.

All participants had no history of neurological or psychiatric disease, alcohol or substance abuse/dependence, and they had no use of psychotropic medications in the past 5 weeks.

### 2.2. Stimuli

Stimuli consisted of 40 faces from the Karolinska Directed Emotional Faces database (Lundqvist et al., 1998). Frontal view photographs of five female and five male faces were selected (females: AF01, AF06, AF13, AF14, and AF15, and males: AM08, AM13, AM17, AM19, and AM20).

Each identity was presented in four emotional expressions: happy (HA), angry (AN), sad (SA), and neutral (NE). To ensure high experimental control, all images were converted into gray-scale images at a resolution of 196 × 265 pixels (3.99° × 5.50° degrees of visual angle seen from 70 cm away), and they were normalized for size and luminance. To prevent potential gender-diagnostic signals, a black oval-shaped mask was used to hide the hair of the faces. Additionally, stimuli with particular facial characteristics that could facilitate gender identification, such as glasses, make-up, moustache and beard, were excluded.

**Figure 1:**
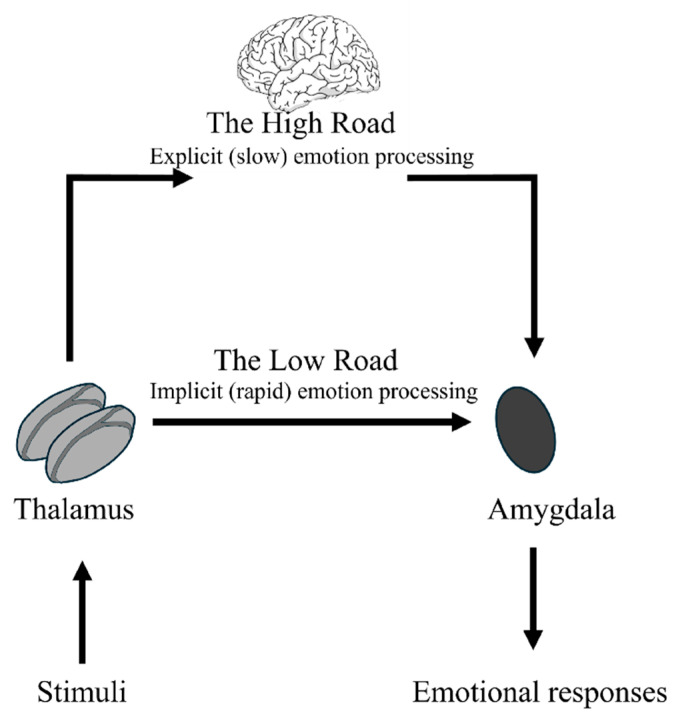
Tracing emotional pathways: the Ledoux model.

**Figure 2:**
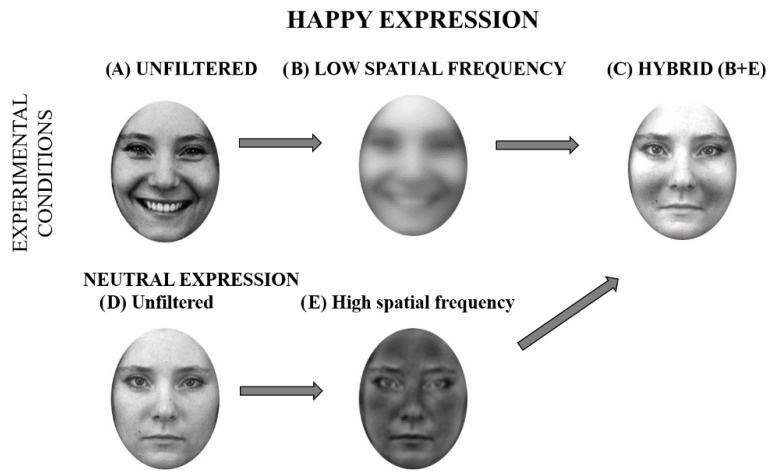
Happy face (**A**) filtered, at a low spatial frequency ((**B**); 1–6 cpi), neutral face (**D**) filtered at a high spatial frequency (7–128 cpi; (**E**)), and happy hybrid face ((**C**) = B + E). The first row represents the happy expressions in each of the three conditions.

The same stimuli were also presented as hybrid faces. Hybrid faces (Figure 1) were created by means of MatLab software (Mathworks Inc., Natick, MA, USA) by overlapping either the happy, angry, or sad expressions filtered at LSFs (1–6 cycles per image, cpi) to the same faces in neutral pose filtered at HSFs (7–128 cpi). Neutral faces were unfiltered or broadband. For the present study, we adapted these validated stimuli (Laeng et al., 2010) by applying the aforementioned oval mask to focus participants’ attention strictly on the internal emotional configuration (see Figure 2). Crucially, each face could be presented in three different positions (LVF, center, and RVF), resulting in a total of 120 unfiltered stimuli and 120 hybrid stimuli (for a grand total of 240 trials).

### 2.3. Procedure

Participants comfortably sat in a dimly lit room and were tested individually at a distance of 70 cm from the computer screen. Participants were provided with written instructions and asked to read them carefully to understand that the experimental procedure consisted of a friendliness judgment task, characterized by the rapid presentation of stimuli. To ensure that the requirements were fully understood and to minimize any potential ambiguity, the experimenter subsequently provided a detailed verbal explanation of these instructions. Participants were instructed to keep their gaze on the central fixation cross for the entire duration of the task and to judge each stimulus (faces) in an isolated manner after its disappearance. All participants, including the control group, used their dominant hand (right hand) to provide responses on the keyboard. Each key corresponds to one of the five levels of the Likert-scale defined as follows: 1 = very unfriendly; 2 = unfriendly; 3 = neutral; 4 = friendly; 5 = very friendly (for the procedure, see e.g., Laeng et al., 2010; Prete et al., 2015a). When the patient R. showed difficulty pressing the response key, she was allowed to give an oral response, and the experimenter pressed the key. Participants were then instructed to keep their gaze on the central fixation cross for the entire duration of the task and to judge each stimulus as quickly and accurately as possible.

Each trial was constituted by a white fixation cross presented in the center of a black screen (ACER, resolution: 1366 × 768 pixels, refresh rate: 60 Hz) for 1000 ms, followed by a stimulus lasting 150 ms. Stimuli could be presented either in the center of the screen or laterally, with the inner side at 214 pixels from the central fixation cross. Subsequently, the screen became black until the participant gave the response.

The entire experimental procedure comprised two blocks separated by one break. A fixed order was employed: during the first block, hybrid faces were presented, and during the second one, unfiltered stimuli were presented. This fixed order was chosen to prevent potential carry-over effects or top-down contamination from the explicit recognition of emotions to the implicit task. By presenting the hybrid (subliminal) stimuli first, we ensured that participants’ judgments were not biased by previous conscious processing of the same face identities in their unfiltered, explicitly emotional versions. Each block was composed of 120 stimuli (10 faces in angry, neutral, sad and happy expressions presented at three different positions), randomly presented, for a total of 240 trials. To allow participants to familiarize themselves with the task and the friendliness scale, seven practice trials were presented before the beginning of the experiment that were not included in the analysis.

The whole experimental procedure was implemented and administered by means of E-prime software (Version 1) (Psychology Software Tools Inc., Pittsburgh, PA, USA) and lasted about 10 min. At the end of the task, participants were asked to complete the Edinburgh Handedness Inventory (Oldfield, 1971), and finally, they were debriefed.

## 3. Results

Statistical analyses were conducted by means of the software Statistica 8.0.550 (StatSoft Inc., Tulsa, OK, USA). To assess the integrity of the experimental effects, we performed within-group exploratory ANOVAs for both the control group and the case study patient separately. Subsequently, to compare the patient’s performance against the control group, we employed specialized single-case statistics (Crawford & Garthwaite, 2002), which are required for clinical comparisons with small normative samples.

Given the specific nature of this single-case study and the small size of the control group (N = 3), we have reported individual performance data to ensure full transparency regarding the behavioral distribution and to allow more precise comparison with the patient’s profile. Table 1 summarizes the mean friendliness ratings for each control participant across all experimental conditions.

### 3.1. Control Group

A repeated-measures ANOVA was carried out, using emotion (happy: HA, neutral: NE, angry: AN and sad: SA), filtering (hybrid and unfiltered) and position (center; left-visual-field, LVF; and right-visual-field, RVF) as within-subjects factors. The friendliness ratings of the control group with each stimulus were used as the dependent variable. Post hoc comparisons were carried out using the Duncan test.

A main effect of emotion was found (F_(3,6)_ = 29.877, MSE = 14.700, *p* < 0.001, η_p_^2^ = 0.94): happy faces (4.00 ± 0.25) were judged as more friendly than neutral (2.58 ± 0.19), sad (2.30 ± 0.17) and angry faces (1.94 ± 0.06; *p* < 0.001, for all comparisons). Moreover, neutral faces were judged as more friendly than sad ones (*p* = 0.040).

The main effects of position (F_(2,4)_ = 2.658, MSE = 0.509, *p* = 0.184, η_p_^2^ = 0.57) and filtering (F_(1,2)_ = 2.685, MSE = 1.474, *p* = 0.243, η_p_^2^ = 0.573) were not significant.

The interaction between emotion and filtering was significant (F_(3,6)_ = 46.419, MSE = 4.195, *p* < 0.001, η_p_^2^ = 0.96). Post hoc comparisons confirmed that in the unfiltered condition, happy faces were judged as more friendly than neutral, sad, and angry faces (*p* < 0.001 for all comparisons). Moreover, neutral unfiltered faces were evaluated as more friendly than sad (*p* < 0.001) and angry (*p* < 0.001) unfiltered faces; unfiltered sad faces were evaluated as more friendly than unfiltered angry faces (*p* = 0.017). In the hybrid condition, happy faces were judged as more friendly than neutral, sad, and angry faces (*p* < 0.002 for all comparisons). Finally, hybrid faces were evaluated as more friendly than unfiltered faces in sad and angry conditions (*p* < 0.001), hybrid faces were evaluated similar to unfiltered faces in neutral conditions (*p* = 0.270), and for happy faces, hybrid faces were evaluated less friendly than unfiltered faces (*p* < 0.001).

The interactions between position and filtering (F_(2,4)_ = 1.658, MSE = 0.165, *p* = 0.298, η_p_^2^ = 0.45), between position and emotion (F_(2,4)_ = 1.519, MSE = 0.136, *p* = 0.252, η_p_^2^ = 0.43) and position, emotion and filtering (F_(6,12)_ = 1.519, MSE = 0.136, *p* = 0.252, η_p_^2^ = 0.43) were not significant.

Although no significant lateralization emerged in the control group, the factor position was purposefully retained as a discrete variable in all statistical models. This allowed us to maintain a one-to-one correspondence between the controls’ performance and the case study patient’s performance in each specific spatial condition. By doing so, we ensured that the subsequent case–control comparisons (using Crawford and Garthwaite’s tests) were executed using a statistically rigorous and spatially aligned baseline, avoiding any potential bias that might arise from averaging across fields in a single-case study design.

### 3.2. Case Study Patient

A repeated-measures ANOVA on patient friendliness ratings on each stimulus as a dependent variable was conducted.

A main effect of filtering was found (F_(1,9)_ = 9.740, MSE = 24.936, *p* = 0.012, η_p_^2^ = 0.52): hybrid faces (2.43 ± 0.14) were judged as more friendly than unfiltered faces (1.79 ± 0.10).

A main effect of emotion was found (F_(3,27)_ = 24.474, MSE = 34.770, *p* < 0.001, η_p_^2^ = 0.73): happy faces (3.22 ± 0.19) were judged as more friendly than neutral (1.99 ± 0.13), sad (1.70 ± 0.08), and angry faces (1.53 ± 0.17; *p* < 0.001, for all comparisons).

The interaction between emotion and filtering was significant (F_(3,27)_ = 3.861, MSE = 3.719, *p* = 0.020, η_p_^2^ = 0.30). Post hoc comparisons confirmed that in both unfiltered and hybrid conditions, happy faces were judged as more friendly than neutral, sad, and angry faces (*p* < 0.007 for all comparisons). Finally, hybrid faces were evaluated as more friendly than unfiltered faces in all expression conditions (*p* < 0.003), except for happy faces, which were evaluated similarly in unfiltered and hybrid conditions (*p* = 0.692).

The main effect of position (F_(2,18)_ = 0.275, MSE = 0.350, *p* = 0.762, η_p_^2^ = 0.03), the interaction between position and filtering (F_(2,18)_ = 2.643, MSE = 2.659, *p* = 0.099, η_p_^2^ = 0.23), and the interaction between position and emotion filtering (F_(6,54)_ = 0.382, MSE = 0.299, *p* = 0.482, η_p_^2^ = 0.09) were not significant.

### 3.3. Case Study Patient Versus the Control Group

The mean scores of the patient were calculated for each condition (four emotional expressions X three positions), for both unfiltered and hybrid stimuli. These means were compared with the 95% confidence intervals computed on the healthy participants’ scores. The results of each condition of the patient were compared with the results of the control group using Crawford and Garthwaite’s (2002) single-case tests. Scores were considered to be significant when the *p*-value was under 0.05 (one-tailed *p*-value). All comparisons were made using a one-tailed level of significance.

**Figure 3:**
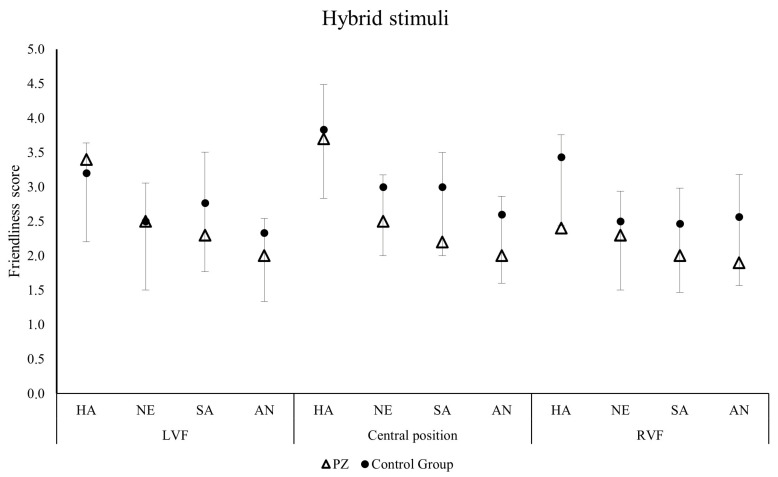
The 95% confidence intervals of friendliness ratings computed on the control group scores (circle), superimposed to the case study patient’s mean ratings (R.’s means are triangle-shaped) in the unfiltered conditions. The figure shows unfiltered happy (HA), neutral (NE), sad (SA) and angry (AN) faces presented in the left visual field (LVF; **left**), in central position (**center**) and in the right visual field (RVF; **right**). The error bars represent the Standard Deviation (SD).

**Figure 4:**
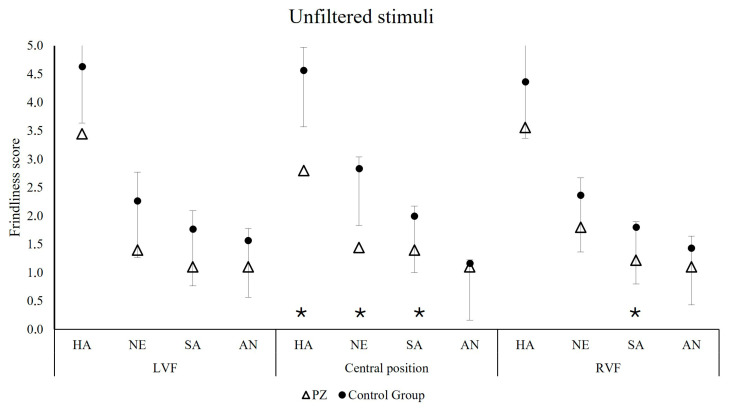
The 95% confidence intervals of friendliness ratings computed on the control group scores (circle), superimposed to the case study patient’s mean ratings (R.’s means are triangle-shaped) in the hybrid conditions. The figure shows hybrid happy (HA), neutral (NE), sad (SA) and angry (AN) faces presented in the left visual field (LVF; **left**), in central position (**center**) and in the right visual field (RVF; **right**). The error bars represent the Standard Deviation (SD). Asterisks show significant comparisons (*p* < 0.050).

The ratings of the patient for unfiltered faces were below the cut-off of the 95% confidence intervals for happy faces presented in central position (t = −3.832, *p* = 0.031), for neutral faces presented in central position (t = −5.732, *p* = 0.014), and for sad faces presented in central (t = −3.057, *p* = 0.046) and right positions (t = −5.023, *p* = 0.019; Figure 3). Importantly, for the hybrid faces, R.’s judgements did not differ from those of the control group (Figure 4). For more details, refer to Table 2.

## 4. Discussion

To better understand emotion processing at both explicit (supraliminal) and implicit (subliminal) levels and the hemispheric asymmetries involved, we investigated the impact of internal capsulo–thalamic damage on implicit and explicit emotion processing, particularly analyzing the performance of a single patient as a key clinical case.

The control group’s performance highlights a clear effect of emotion on friendliness judgments, an effect that is significantly modulated by the filtering type employed (hybrid stimuli). In the explicit evaluation condition (unfiltered faces), friendliness judgments follow a precise hierarchy: happy faces are judged as the friendliest, followed by neutral faces (perceived as more friendly than sad and angry), then sad faces (perceived as more friendly than angry) and lastly angry faces. This finding suggests an ability to discriminate emotional valences under explicit processing conditions. Conversely, in the implicit evaluation condition (hybrid faces), although happy hybrid faces retain their preeminence in perceived friendliness, the results show no significant differences between negative emotions (sad and angry) and the neutral ones.

However, results in the hybrid condition confirmed that the hidden emotional content conveyed by the LSFs of faces is processed at a subliminal level (Mermillod et al., 2010; Laeng et al., 2010, 2013); however, this processing may not be sufficient to distinguish subtle nuances of negative valence. Our findings are partially in contrast with the literature on the younger population. While some studies on the young adult population demonstrate a differentiation in emotional judgments across a full spectrum of emotions (e.g., Prete et al., 2014), others show effects limited to the distinction between positive and negative valence, often using hybrid or low-spatial-frequency faces (e.g., Tommasi et al., 2021). Moreover, our findings may be a direct consequence of the neurobiological changes associated with aging. In fact, the ability for context-sensitive processing mediated by LSFs declines with aging. The thalamus undergoes modifications with age, and its vulnerability may impact the magnocellular pathway, which is crucial for the rapid, automatic processing of LSFs (Wang et al., 2019). This may explain why, in our control group, the implicit processing of LSFs seems to be sufficient to differentiate positive from negative/neutral stimuli, but not to distinguish between different negative emotions (e.g., sadness vs angry).

This evidence is in line with Loughead and colleagues (2008), who showed reduced activation of the thalamus when happy faces were presented, and an opposite pattern when angry faces were presented, together with reduced activation of the middle frontal gyrus when sad faces were presented. Taken together, these results highlight the crucial role of the thalamus in emotion processing, particularly in the processing of cues conveying an imminent threat to survival (Loughead et al., 2008).

The failure of older adults to discriminate between negative emotions in the implicit (LSF) condition may reflect both structural and functional age-related changes.

The fact that the patient differs from controls specifically in the explicit condition, while showing similar performance in the hybrid condition, provides crucial evidence that internal capsule damage prevents the cortex from receiving the necessary detailed information. This is consistent with the hypothesis that the ALIC and PLIC are associated with attentional processes (Niogi et al., 2010) related to the processing of anger and sadness.

Structural MRI studies (Raz, 2000) reveal modest age-associated volumetric reductions in limbic regions, including the hippocampus, parahippocampal gyrus, and amygdala. This reduced structural integrity of these subcortical regions may compromise the functional connectivity and, thus, the rapid and automatic processing of negative emotions (Hrybouski et al., 2016).

**Figure 5:**
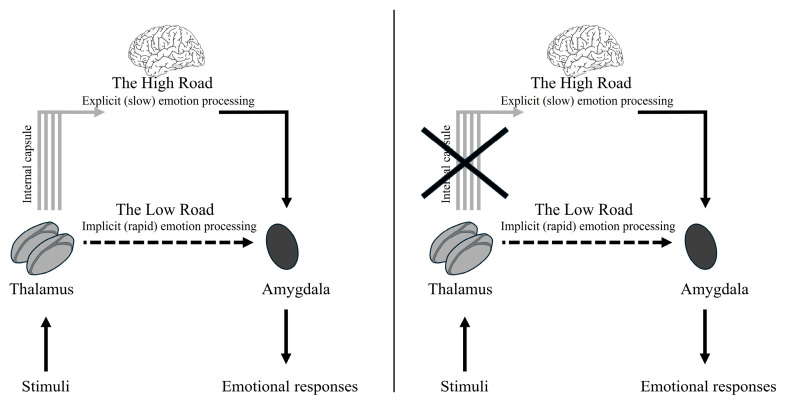
LeDoux’s dual-route model of emotion processing in older adults. Left: Schematic representation of LeDoux’s model; the four lines represent the internal capsule, containing thalamo–cortical projection fibers crucial for the “high road.” The dashed line for the “low road” suggests a potential weakening in older adults. Right: Schematic representation of the “high road” (cortical, explicit, slow) and “low road” (subcortical, implicit, rapid) pathways to the amygdala, with an indication of potential disruption by internal capsule damage.

The finer discrimination between negative emotions emerges in the explicit condition, which involves unfiltered stimulus processing by the cortical pathway. Such fine discrimination requires a more detailed analysis of facial features, which is typically handled by the cortical pathway (Figure 5). This implies that conscious, explicit processing can partially compensate for the age-related decline in early, automatic emotional processing. The study by Prete et al. (2024) shows that in healthy older adults, the cortical pathway (unfiltered) ensures the best performance and allows for the discrimination of anger. In the current study, the fact that the case study patient differs from the controls specifically in this condition, while showing similar performance in the hybrid condition, provides crucial evidence that internal capsule damage prevents the cortex from receiving the necessary detailed information. While healthy older adults in Prete et al. (2024) use the cortical pathway to compensate for potential decline, the case study patient cannot do so due to this physical disconnection.

This evidence aligns with Smith and Schyns (2009) who suggested that happiness, unlike anger, represents a “distal” emotion relying on LSF processing and thus is perceivable even with less detailed visual information. Prete et al. (2024) confirmed this hierarchy: happiness scores were significantly higher than anger or happiness alone, and performance degraded linearly from unfiltered to hybrid stimuli. This reinforces the conclusion that the subcortical route serves as a basis for a rapid, fundamental discrimination between “good” (positive/happy) and “bad” (negative/unpleasant) stimuli, a mechanism essential for survival. This could explain why, in our healthy sample, only hybrid happy faces were distinguished from neutral faces. This finding is consistent with the “coarse-to-fine hypothesis,” according to which the brain initially uses LSFs for a rapid global percept, and then engages HSFs for detailed analysis.

Supporting this framework, the literature indicates that non-conscious processing relies more heavily on LSF and occurs significantly faster than conscious evaluation, suggesting that different neural pathways are recruited based on the degree of awareness (Willenbockel et al., 2012). Recent iEEG data further confirms this by identifying a rapid amygdala response, specifically in the low gamma band, for LSFs. Crucially, this rapid subcortical activity is present even when stimuli are invisible and is notably absent in cortical regions such as the fusiform gyrus, providing direct evidence for a subcortical pathway that operates independently of conscious awareness and cortical influence (Wang et al., 2023). Consequently, in situations requiring implicit LSF-based evaluation, middle-aged adults might rely more on a global processing of valence, mediated by relatively preserved subcortical circuits. This means that they can generally identify the difference between positive and negative emotions as a broad category. This general (superficial) capacity is likely to be mediated by relatively preserved subcortical circuits. However, this global processing is not sufficient to discriminate between emotions within the same valence (e.g., sadness from anger, both being negative).

### 4.1. Direct Comparison and Clinical Evidence

A direct comparison between the case study patient and the control group reveals no significant differences in the implicit evaluation condition, suggesting that the case study patient’s automatic emotional processing mechanisms are likely preserved. Conversely, significant differences appear in the explicit evaluation condition, in which the case study patient showed significantly different friendliness judgments than the control group.

Cheung et al. (2006) showed that localized basal ganglia damage produced a decrease in anger, disgust and fear recognition, whereas thalamic damage impaired sadness recognition. In our patient, the impairment of negative emotion recognition in the explicit condition might be due to the interruption of information flow caused by the disruption of the internal capsule (IC). A damaged IC could cause a disconnection of distant regions or mild damage in the nearby thalamus and striatum. In fact, the ALIC contains fiber tracts involved in emotion and cognition (Sprengelmeyer et al., 1996; Pauli et al., 2016).

Another possible explanation may be related to the presentation modality. Recent MRI studies highlighted an involvement of the internal capsule in attentional shifting (Sullivan et al., 2010) and alerting (Niogi et al., 2010). Thus, when emotional stimuli are presented in an explicit fashion and require attentional processes, the activity of the internal capsule (Niogi et al., 2010) should be necessary to correctly process the emotional content. However, when the same stimuli are presented in an implicit fashion (i.e., hybrids), they do not require specific attentional processes, and the internal capsule damage would not affect emotion processing. This hypothesis is consistent with Pessoa et al. (2002), who found a modulation of emotion processing depending on attentional control processes. Indeed, in this condition, the case study patient also differentiated between positive and negative emotions but not between negative emotions. Conversely, the control group made significantly different judgements according to the emotions expressed by the faces. These specific discrepancies may indicate that the case study patient’s lesion influences her performance in the explicit condition, impairing the ability to accurately distinguish between negative emotions. These results suggest that explicit emotion processing relies on intact capsulo–thalamic connections, in accordance with the model proposed by LeDoux (2003). Specifically, the performance of our patient suggests an involvement of the internal capsule in explicit emotion processing and supports the critical role of thalamo–cortical projections in explicit emotion processing. Damage to the projections connecting the thalamus to the cortex (Figure 5) could alter the ability to distinguish between different emotions with the same valence (e.g., sadness and anger).

### 4.2. Hemispheric Specialization and Aging

This study also aimed to verify hemispheric lateralization in the processing of LSFs using the patient with a right capsular lesion. Contrary to expectations based on the scientific literature, suggesting a right specialization in holistic/LSF processing (Peyrin et al., 2003; Goffaux et al., 2005; Gable et al., 2013; Proverbio & Zani, 2021), the case study patient showed normal performance in recognizing hybrid faces (which rely on LSFs), but displayed a specific deficit for explicit (unfiltered) faces. This clinical dissociation suggests that the relationship between the right hemisphere and holistic processing is not rigid. If the processing of LSFs depends mainly on the cortex (Gable et al., 2013; Proverbio & Zani, 2021), then the case study patient should have shown a deficit in the hybrid condition. The fact that her performance in the hybrid condition was not compromised, despite having a lesion that disrupts connections to the cortex, supports the hypothesis that these intact subcortical structures are sufficient to ensure global/LSF perception. However, we cannot conclude about hemispheric lateralization. The absence of a deficit in LSF processing could be due to the integrity of the right subcortical structures, a bilateral function of those structures, or compensation from the left hemisphere. The clinical case demonstrates that the role of subcortical pathways in emotional processing is a valid hypothesis and worthy of further investigation. This observation is consistent with the findings of Prete et al. (2024), which do not support the theory of a generalized decline in subcortical emotional activity during aging, as evidenced by stable performance in LSFs and hybrid conditions across age groups. Instead, their results partially confirm that while the cortical route is preserved and used for detailed top-down compensation in healthy subjects, the subcortical path continues to provide a reliable baseline for early emotional detection.

Regarding hemispheric specialization, the so-called “Right Hemisphere Hypothesis” argues that the right hemisphere is specialized in processing all emotions regardless of emotional valence (Gainotti, 1972, 2005). Several studies have shown that tachistoscopic lateralized presentation of emotional stimuli in the LVF induces better discrimination than the presentation in the RVF (Landis et al., 1979; Ley & Bryden, 1979). On the other hand, the “Valence Hypothesis” posits that the right hemisphere is specialized in processing negative emotions, whereas the left hemisphere is specialized in processing positive emotions (Davidson et al., 1987; Ehrlichman, 1987). Our results did not confirm either of these two hypotheses, suggesting an absence of hemispheric asymmetries in emotional processing.

According to the HAROLD model (Hemispheric Asymmetry Reduction in OLder adults; Cabeza, 2002), cognitive functions become less lateralized in the brain with increasing age and, to compensate for this reduction in lateralization, an additional network is activated with compensatory function. This hypothesis argues that cerebral differences will be reduced according to time function and a decrease in functionality will not be equal in the two hemispheres: a different aging for the two hemispheres occurs (right more than the left). This evidence is in line with our results showing no significant differences between RVF and LVF presentation, suggesting a reduced hemispheric specialization.

Further consideration concerns potential perceptual lateralization effects. While right-sided brain damage is frequently associated with impairments in perceiving stimuli in the left visual field (LVF) due to spatial neglect or specific right-hemispheric specialization for face processing, patient R. did not show a significantly lower performance for stimuli presented in the LVF compared with those in the right visual field (RVF) or central position. Although an exploratory ANOVA is not the most appropriate statistical tool for a single-case study, its use allowed us to observe the patient’s data trend, which clearly showed no lateralization effects. Taken together with the data from the control group, the findings suggest two main points: first, it confirms a general reduction in hemispheric specialization associated with aging (consistent with the HAROLD model), and second, it indicates that R.’s specific impairment in evaluating friendliness, especially for negative emotions, cannot be attributed to a primary perceptual deficit or spatial neglect, but rather points toward a deficit in the emotional evaluation of the stimulus itself.

Regarding the brain lateralization pattern of facial emotion recognition, it has been suggested that the right hemisphere would be more involved in emotional processing than the left hemisphere (Bradshaw & Nettleton, 1981), and this hypothesis seems to be linked to a right hemispheric specialization in holistic processing, allowing us to process the complex visual patterns of emotional input (Cheung et al., 2006). The right-hemispheric superiority is also evident in studies conducted with split-brain patients (Prete et al., 2015b; Prete & Tommasi, 2018). Split-brain patients do not appear to have difficulties in emotional face recognition when stimuli are presented to the right hemisphere (Benowitz et al., 1983), failing instead when the same stimuli are presented to the left hemisphere. Crucially, we did not observe any significant lateralization effect on the recognition of happy, neutral, sad, or angry faces in the control group. Maintaining the position factor throughout the analysis, despite its lack of significance, was fundamental for the validity of our single-case study. It confirmed that the control group provided a stable, non-lateralized baseline against which the patient’s performance could be compared, condition by condition, ensuring a rigorous, spatially aligned comparison (Crawford & Garthwaite, 2002). This approach demonstrates that the patient’s deficits in explicit emotion processing are specifically related to the disruption of thalamo–cortical connectivity rather than a generalized loss of hemispheric advantage that could have been masked by collapsing the data.

## 5. Limitations and Future Directions

Despite the clinical and theoretical relevance of our findings, some limitations must be acknowledged. First, as this is a single-case study, the generalizability of the results is inherently limited. While patient R. provides a unique opportunity to observe the effects of a focal internal capsule–thalamic lesion, individual variability in neural plasticity and brain reorganization following damage could influence the observed behavioral patterns. Second, our control group, although carefully matched, consisted of a small number of participants (N en = 3). While we addressed this by providing individual data points to ensure transparency, future studies should involve larger samples to more robustly characterize age-related changes in spatial frequency processing. Future research incorporating neuroimaging or physiological measures could provide further insight into the subcortical activation patterns during implicit emotional evaluation in patients with similar disconnections.

## 6. Conclusions

To conclude, the present study suggests an involvement of the internal capsule in explicit emotion processing, but also reveals a preserved ability to process implicit emotional stimuli when this structure is damaged. This conclusion is in line with the existence of a double route for emotion detection (i.e., LeDoux, 1996; Gainotti, 2012), suggesting that the internal capsule would take part in the slow cortical route of emotion detection.

The clinical dissociation between explicit and implicit emotional processing in the patient suggests that the relationship between the right hemisphere and holistic processing is not rigid and is more complex than we think.

## Figures and Tables

**Table 1:** Individual performances of the control group. The table displays the mean friendliness ratings (on a scale from 1 to 5 for each of the three control participants (pp1, pp2, pp3). Data are presented separately for the explicit task (unfiltered stimuli = Nf) and the implicit task (hybrid stimuli = Hy) for each emotional expression (happy = HA, neutral = NE, sad = SA and angry = AN). Legend: 1 = LVF, 2 = Center and 3 = RVF.

		1_HA	1_NE	1_SA	1_AN	2_HA	2_NE	2_SA	2_AN	3_HA	3_NE	3_SA	3_AN
pp_1	HY	2.9	2.6	2.5	2.1	3.2	3.1	3.00	2.4	3.3	2.8	2.6	3.1
pp_2	Hy	3.7	300	3.6	2.4	3.8	3.1	3.5	2.9	3.8	2.7	2.9	2.7
pp_3	Hy	3.00	1.9	2.2	2.5	4.5	2.8	2.5	2.5	3.2	2.00	1.9	1.9
pp_1	Nf	3.9	2.2	1.4	1.5	4.2	3.00	1.8	1.2	3.6	2.7	1.7	1.5
pp_2	Nf	5.00	2.8	1.9	1.4	4.5	2.9	2.1	1.2	4.5	2.3	1.8	1.6
pp_3	Nf	5.00	1.8	2.00	1.8	5.00	2.6	2.1	1.1	5.00	2.1	1.9	1.2
Hy	Mean	3.2	2.5	2.77	2.33	3.83	3.00	3.00	2.6	3.43	2.5	2.47	2.57
Hy	S.D.	0.44	0.56	0.74	0.21	0.65	0.17	0.5	0.26	0.32	0.44	0.51	0.61
Nf	Mean	4.63	2.27	1.77	1.57	4.57	2.83	2.00	1.17	4.37	2.37	1.8	1.43
Nf	S.D.	0.64	0.5	0.32	0.21	0.4	0.21	0.17	0.06	0.71	0.31	0.1	0.21

**Table 2:** T-values and one-tailed probabilities for the comparison of the patient’s friendliness ratings against the control group’s mean for each emotion, position, and filtering condition. Significant differences are highlighted in blue.

Filtering	Position	Emotion	t Value	One-Tailed Probability	95% Lower	95% Upper	Effect Size (Z-cc)	From	To
Hybrid	LVF	HA	0.394	0.366	21.35%	94.68%	0.455	−0.794	1.615
		NE	0.000	0.500	12.89%	87.10%	0.000	−1.132	1.132
		SA	−0.550	0.319	3.21%	75.46%	−0.635	−1.851	0.689
		AN	−1.361	0.153	0.04%	61.14%	−1.571	−3.346	0.283
	Central position	HA	−0.173	0.439	9.27%	83.35%	−0.200	−1.324	0.968
		NE	−2.547	0.062	0.00%	45.18%	−2.941	−5.833	−0.121
		SA	−1.386	0.150	0.03%	60.76%	−1.600	−3.395	0.273
		AN	−1.925	0.097	0.00%	52.97%	−2.308	−4.664	0.050
	RVF	HA	−2.788	0.054	0.00%	42.46%	−3.219	−6.351	−0.190
		NE	−0.394	0.366	5.32%	78.65%	−0.455	−1.615	0.794
		SA	−0.798	0.254	1.16%	70.70%	−0.922	−2.270	0.545
		AN	−0.951	0.221	0.54%	67.94%	−1.098	−2.549	0.466
Unfiltered	LVF	HA	−1.610	0.124	0.01%	57.38%	−1.859	−3.853	0.186
		NE	−1.507	0.135	0.014%	58.91%	−1.740	−3.641	0.225
		SA	−1.813	0.106	0.00%	54.49%	−2.094	−4.275	0.113
		AN	−1.938	0.096	0.00%	52.79%	−2.238	−4.537	0.070
	Central position	HA	−3.832	0.031	0.00%	32.19%	−4.425	−8.623	−0.426
		NE	−5.732	0.014	0.00%	18.60%	−6.619	−12.769	−0.893
		SA	−3.057	0.046	0.00%	39.59%	−3.529	−6.933	−0.264
		AN	−1.010	0.209	0.39%	66.91%	−1.167	−2.660	0.437
	RVF	HA	−0.988	0.214	0.44%	67.29%	−1.141	−2.618	0.448
		NE	−1.592	0.126	0.01%	57.64%	−1.839	−3.817	0.193
		SA	−5.023	0.019	0.00%	23.00%	−5.800	−11.235	−0.739
		AN	−1.429	0.145	0.025%	60.09%	−1.571	−3.346	0.283

## Data Availability

The data that support the findings of this study are available upon request from the corresponding author. The data are not publicly available because they contain information that could compromise the privacy of patients and research participants.

## Abbreviations

The following abbreviations are used in this manuscript:

LSF	Low Spatial Frequency
HSF	High spatial frequency
LVF	Left visual field
RVF	Right visual field
Ha	Happy faces
Ne	Neutral faces
Sa	Sad faces
An	Angry faces

## References

[B1-jintelligence-14-00060] Adolphs R., Tranel D., Damasio H., Damasio A. (1994). Impaired recognition of emotion in facial expressions following bilateral damage to the human amygdala. Nature.

[B2-jintelligence-14-00060] Axelrod V., Bar M., Rees G. (2015). Exploring the unconscious using faces. Trends in Cognitive Sciences.

[B3-jintelligence-14-00060] Bar M., Neta M. (2006). Humans prefer curved visual objects. Psychological Science.

[B4-jintelligence-14-00060] Beck A. T., Steer R. A., Ball R., Ranieri W. F. (1996). Comparison of beck depression inventories-IA and-II in psychiatric outpatients. Journal of Personality Assessment.

[B5-jintelligence-14-00060] Benowitz L. I., Bear D. M., Rosenthal R., Mesulam M. M., Zaidel E., Sperry R. W. (1983). Hemispheric specialization in nonverbal communication. Cortex.

[B6-jintelligence-14-00060] Bradshaw J. L., Nettleton N. C. (1981). The nature of hemispheric specialization in man. Behavioral and Brain Sciences.

[B7-jintelligence-14-00060] Brooks S. J., Savov V., Allzén E., Benedict C., Fredriksson R., Schiöth H. B. (2012). Exposure to subliminal arousing stimuli induces robust activation in the amygdala, hippocampus, anterior cingulate, insular cortex and primary visual cortex: A systematic meta-analysis of fMRI studies. NeuroImage.

[B8-jintelligence-14-00060] Cabeza R. (2002). Hemispheric asymmetry reduction in older adults: The HAROLD model. Psychology and Aging.

[B9-jintelligence-14-00060] Campbell F. W., Robson J. G. (1968). Application of Fourier analysis to the visibility of gratings. The Journal of Physiology.

[B10-jintelligence-14-00060] Celeghin A., Mazzoni N., Mattavelli G. (2020). Explicit and implicit emotion processing: Neural basis, perceptual and cognitive mechanisms. Frontiers in Psychology.

[B11-jintelligence-14-00060] Cheung C. C., Lee T. M., Yip J. T., King K. E., Li L. S. (2006). The differential effects of thalamus and basal ganglia on facial emotion recognition. Brain and Cognition.

[B12-jintelligence-14-00060] Christmas D., Eljamel M. S., Butler S., Hazari H., MacVicar R., Steele J. D., Livingstone A., Matthews K. (2011). Long term outcome of thermal anterior capsulotomy for chronic, treatment refractory depression. Journal of Neurology, Neurosurgery & Psychiatry.

[B13-jintelligence-14-00060] Coenen V. A., Panksepp J., Hurwitz T. A., Urbach H., Mädler B. (2012). Human medial forebrain bundle (MFB) and anterior thalamic radiation (ATR): Imaging of two major subcortical pathways and the dynamic balance of opposite affects in understanding depression. The Journal of Neuropsychiatry and Clinical Neurosciences.

[B14-jintelligence-14-00060] Crawford J. R., Garthwaite P. H. (2002). Investigation of the single case in neuropsychology: Confidence limits on the abnormality of test scores and test score differences. Neuropsychologia.

[B15-jintelligence-14-00060] Cunningham W. A., Johnson M. K., Raye C. L., Gatenby J. C., Gore J. C., Banaji M. R. (2004). Separable neural components in the processing of black and white faces. Psychological Science.

[B16-jintelligence-14-00060] Darwin C. (1872). The expression of emotions in man and animals.

[B17-jintelligence-14-00060] Davidson R. J., Mednick D., Moss E., Saron C., Schaffer C. E. (1987). Ratings of emotion in faces are influenced by the visual field to which stimuli are presented. Brain and Cognition.

[B18-jintelligence-14-00060] de Gelder B., van Honk J., Tamietto M. (2011). Emotion in the brain: Of low roads, high roads and roads less travelled. Nature Reviews Neuroscience.

[B19-jintelligence-14-00060] Diano M., Celeghin A., Bagnis A., Tamietto M. (2017). Amygdala response to emotional stimuli without awareness: Facts and interpretations. Frontiers in Psychology.

[B20-jintelligence-14-00060] Ehrlichman H. (1987). Hemispheric asymmetry and positive-negative affect. Duality and unity of the brain.

[B21-jintelligence-14-00060] Emos M. C., Agarwal S. (2019). Neuroanatomy, internal capsule. StatPearls [Internet].

[B22-jintelligence-14-00060] Fimm B., Zahn R., Mull M., Kemeny S., Buchwald F., Block F., Schwarz M. (2001). Asymmetries of visual attention after circumscribed subcortical vascular lesions. Journal of Neurology, Neurosurgery and Psychiatry.

[B23-jintelligence-14-00060] Gable P. A., Poole B. D., Cook M. S. (2013). Asymmetrical hemisphere activation enhances global–local processing. Brain and Cognition.

[B24-jintelligence-14-00060] Gainotti G. (1972). Emotional behavior and hemispheric side of the lesion. Cortex.

[B25-jintelligence-14-00060] Gainotti G. (2005). Emotions, unconscious processes, and the right hemisphere. Neuropsychoanalysis.

[B26-jintelligence-14-00060] Gainotti G. (2012). Unconscious processing of emotions and the right hemisphere. Neuropsychologia.

[B27-jintelligence-14-00060] Garvert M. M., Friston K. J., Dolan R. J., Garrido M. I. (2014). Subcortical amygdala pathways enable rapid face processing. NeuroImage.

[B28-jintelligence-14-00060] Goffaux V., Hault B., Michel C., Vuong Q. C., Rossion B. (2005). The respective role of low and high spatial frequencies in supporting configural and featural processing of faces. Perception.

[B29-jintelligence-14-00060] Hrybouski S., Aghamohammadi-Sereshki A., Madan C. R., Shafer A. T., Baron C. A., Seres P., Beaulieu C., Olsen F., Malykhin N. V. (2016). Amygdala subnuclei response and connectivity during emotional processing. Neuroimage.

[B30-jintelligence-14-00060] Johnson M. H. (2005). Subcortical face processing. Nature Reviews Neuroscience.

[B31-jintelligence-14-00060] Juruena M. F., Giampietro V. P., Smith S. D., Surguladze S. A., Dalton J. A., Benson P. J., Cleare A. J., Fu C. H. (2010). Amygdala activation to masked happy facial expressions. Journal of the International Neuropsychological Society.

[B32-jintelligence-14-00060] Laeng B., Profeti I., Saether L., Adolfsdottir S., Lundervold A. J., Vangberg T., Øvervoll M., Johnse S. H., Waterloo K. (2010). Invisible expressions evoke core impressions. Emotion.

[B33-jintelligence-14-00060] Laeng B., Sæther L., Holmlund T., Wang C. E. A., Waterloo K., Eisemann M., Halvorsen M. (2013). Invisible emotional expressions influence social judgments and pupillary responses of both depressed and non-depressed individuals. Frontiers in Psychology.

[B34-jintelligence-14-00060] Landis T., Assal G., Perret E. (1979). Opposite cerebral hemispheric superiorities for visual associative processing of emotional facial expressions and objects. Nature.

[B35-jintelligence-14-00060] LeDoux J. (1996). The emotional brain: The mysterious underpinnings of emotional life.

[B36-jintelligence-14-00060] LeDoux J. (2003). The emotional brain, fear, and the amygdala. Cellular and Molecular Neurobiology.

[B37-jintelligence-14-00060] LeDoux J. (2012). Rethinking the emotional brain. Neuron.

[B38-jintelligence-14-00060] Le Grand R. L., Mondloch C. J., Maurer D., Brent H. P. (2004). Impairment in holistic face processing following early visual deprivation. Psychological Science.

[B39-jintelligence-14-00060] Levy J., Heller W., Banich M. T., Burton L. A. (1983). Asymmetry of perception in free viewing of chimeric faces. Brain and Cognition.

[B40-jintelligence-14-00060] Ley R. G., Bryden M. P. (1979). Hemispheric differences in processing emotions and faces. Brain and Language.

[B41-jintelligence-14-00060] Loughead J., Gur R. C., Elliott M., Gur R. E. (2008). Neural circuitry for accurate identification of facial emotions. Brain Research.

[B42-jintelligence-14-00060] Lundqvist D., Flykt A., Öhman A. (1998). The Karolinska directed emotional faces (KDEF). CD ROM from Department of Clinical Neuroscience, Psychology Section, Karolinska Institutet.

[B43-jintelligence-14-00060] Machado A., Haber S., Sears N., Greenberg B., Malone D., Rezai A. (2009). Functional topography of the ventral striatum and anterior limb of the internal capsule determined by electrical stimulation of awake patients. Clinical Neurophysiology.

[B44-jintelligence-14-00060] McFadyen J., Mattingley J. B., Garrido M. I. (2019). An afferent white matter pathway from the pulvinar to the amygdala facilitates fear recognition. Elife.

[B45-jintelligence-14-00060] Mermillod M., Droit-Volet S., Devaux D., Schaefer A., Vermeulen N. (2010). Are coarse scales sufficient for fast detection of visual threat?. Psychological Science.

[B46-jintelligence-14-00060] Méndez-Bértolo C., Moratti S., Toledano R., Lopez-Sosa F., Martínez-Alvarez R., Mah Y. H., Vuilleumier P., Gil-Nagel A., Strange B. A. (2016). A fast pathway for fear in human amygdala. Nature Neuroscience.

[B47-jintelligence-14-00060] Mithani K., Davison B., Meng Y., Lipsman N. (2020). The anterior limb of the internal capsule: Anatomy, function, and dysfunction. Behavioural Brain Research.

[B48-jintelligence-14-00060] Narumoto J., Yamada H., Iidaka T., Sadato N., Fukui K., Itoh H., Yonekura Y. (2000). Brain regions involved in verbal or non-verbal aspects of facial emotion recognition. Neuroreport.

[B49-jintelligence-14-00060] Niogi S. N., Mukherjee P., Ghajar J., McCandliss B. D. (2010). Individual differences in distinct components of attention are linked to anatomical variations in distinct white matter tracts. Frontiers in Neuroanatomy.

[B50-jintelligence-14-00060] Okun M. S., Mann G., Foote K. D., Shapira N. A., Bowers D., Springer U., Knight W., Martin P., Goodman W. K. (2007). Deep brain stimulation in the internal capsule and nucleus accumbens region: Responses observed during active and sham programming. Journal of Neurology, Neurosurgery & Psychiatry.

[B51-jintelligence-14-00060] Oldfield R. C. (1971). Edinburgh handedness inventory. Journal of Abnormal Psychology.

[B52-jintelligence-14-00060] Öhman A., Mineka S. (2001). Fears, phobias, and preparedness: Toward an evolved module of fear and fear learning. Psychological Review.

[B53-jintelligence-14-00060] Papez J. W. (1937). A proposed mechanism of emotion. Archives of Neurology & Psychiatry.

[B54-jintelligence-14-00060] Pauli W. M., O’Reilly R. C., Yarkoni T., Wager T. D. (2016). Regional specialization within the human striatum for diverse psychological functions. Proceedings of the National Academy of Sciences.

[B55-jintelligence-14-00060] Pessoa L., Adolphs R. (2010). Emotion processing and the amygdala: From a ‘low road’ to ‘many roads’ of evaluating biological significance. Nature Reviews Neuroscience.

[B56-jintelligence-14-00060] Pessoa L., McKenna M., Gutierrez E., Ungerleider L. G. (2002). Neural processing of emotional faces requires attention. Proceedings of the National Academy of Sciences.

[B57-jintelligence-14-00060] Peyrin C., Chauvin A., Chokron S., Marendaz C. (2003). Hemispheric specialization for spatial frequency processing in the analysis of natural scenes. Brain and Cognition.

[B58-jintelligence-14-00060] Prete G., Capotosto P., Zappasodi F., Laeng L., Tommasi L. (2015a). The cerebral correlates of subliminal emotions: An electroencephalographic study with emotional hybrid faces. European Journal of Neuroscience.

[B59-jintelligence-14-00060] Prete G., Ceccato I., Bartolini E., Di Crosta A., La Malva P., Palumbo R., Laeng B., Tommasi L., Mammarella N., Di Domenico A. (2024). Detecting implicit and explicit facial emotions at different ages. European Journal of Ageing.

[B60-jintelligence-14-00060] Prete G., D’Ascenzo S., Laeng B., Fabri M., Foschi N., Tommasi L. (2015b). Conscious and unconscious processing of facial expressions: Evidence from two split-brain patients. Journal of Neuropsychology.

[B61-jintelligence-14-00060] Prete G., Laeng B., Tommasi L. (2014). Lateralized hybrid faces: Evidence of a valence-specific bias in the processing of implicit emotions. Laterality: Asymmetries of Body, Brain and Cognition.

[B62-jintelligence-14-00060] Prete G., Laeng B., Tommasi L. (2018). Modulating adaptation to emotional faces by spatial frequency filtering. Psychological Research.

[B63-jintelligence-14-00060] Prete G., Tommasi L. (2018). Split-brain patients: Visual biases for faces. Progress in Brain Research.

[B64-jintelligence-14-00060] Proverbio A. M., Zani A. (2021). Hemispheric asymmetry in visual processing: An ERP study on spatial frequency gratings. Symmetry.

[B65-jintelligence-14-00060] Proverbio A. M., Zani A., Avella C. (1997). Hemispheric asymmetries for spatial frequency discrimination in a selective attention task. Brain and Cognition.

[B66-jintelligence-14-00060] Raz N., Craik F. I. M., Salthouse T. A. (2000). Aging of the brain and its impact on cognitive performance: Integration of structural and functional findings. The handbook of aging and cognition.

[B67-jintelligence-14-00060] Safadi Z., Grisot G., Jbabdi S., Behrens T. E., Heilbronner S. R., McLaughlin N. C., Mandeville J., Versace A., Phillips M. L., Lehman J. F., Yendiki A., Yendiki A. (2018). Functional segmentation of the anterior limb of the internal capsule: Linking white matter abnormalities to specific connections. Journal of Neuroscience.

[B68-jintelligence-14-00060] Schyns P. G., Oliva A. (1999). Dr. Angry and Mr. Smile: When categorization flexibly modifies the perception of faces in rapid visual presentations. Cognition.

[B69-jintelligence-14-00060] Seligman M. E. (1971). Phobias and preparedness. Behavior Therapy.

[B70-jintelligence-14-00060] Sergent J. (1982). The cerebral balance of power: Confrontation or cooperation?. Journal of Experimental Psychology: Human Perception and Performance.

[B71-jintelligence-14-00060] Sergent J. (1985). Influence of task and input factors on hemispheric involvement in face processing. Journal of Experimental Psychology: Human Perception and Performance.

[B72-jintelligence-14-00060] Smith F. W., Schyns P. G. (2009). Smile through your fear and sadness: Transmitting and identifying facial expression signals over a range of viewing distances. Psychological Science.

[B73-jintelligence-14-00060] Sprengelmeyer R., Young A. W., Calder A. J., Karnat A., Lange H., Hömberg V., Perrett D. I., Rowland D. (1996). Loss of disgust: Perception of faces and emotions in Huntington’s disease. Brain.

[B74-jintelligence-14-00060] Sullivan E. V., Zahr N. M., Rohlfing T., Pfefferbaum A. (2010). Fiber tracking functionally distinct components of the internal capsule. Neuropsychologia.

[B75-jintelligence-14-00060] Tamietto M., De Gelder B. (2010). Neural bases of the non-conscious perception of emotional signals. Nature Reviews Neuroscience.

[B76-jintelligence-14-00060] Tippett D. C., Godin B. R., Oishi K., Oishi K., Davis C., Gomez Y., Trupe L. A., Kim E. H., Hillis A. E. (2018). Impaired recognition of emotional faces after stroke involving right amygdala or insula. Seminars in speech and language.

[B77-jintelligence-14-00060] Tommasi V., Prete G., Tommasi L. (2021). The role of low spatial frequencies in facial emotion processing: A study on anorthoscopic perception. Visual Cognition.

[B78-jintelligence-14-00060] Vuilleumier P., Armony J. L., Driver J., Dolan R. J. (2003). Distinct spatial frequency sensitivities for processing faces and emotional expressions. Nature Neuroscience.

[B79-jintelligence-14-00060] Wang Y., Luo L., Chen G., Luan G., Wang X., Wang Q., Fang F. (2023). Rapid processing of invisible fearful faces in the human amygdala. Journal of Neuroscience.

[B80-jintelligence-14-00060] Wang Y., Xu Q., Luo J., Hu M., Zuo C. (2019). Effects of age and sex on subcortical volumes. Frontiers in Aging Neuroscience.

[B81-jintelligence-14-00060] Willenbockel V., Lepore F., Nguyen D. K., Bouthillier A., Gosselin F. (2012). Spatial frequency tuning during the conscious and non-conscious perception of emotional facial expressions–an intracranial ERP study. Frontiers in Psychology.

[B82-jintelligence-14-00060] Yip J. T., Leung K. K., Li L. S., Lee T. M. (2004). The role of sub-cortical brain structures in emotion recognition. Brain Injury.

[B83-jintelligence-14-00060] Zald D. H. (2003). The human amygdala and the emotional evaluation of sensory stimuli. Brain Research Reviews.

